# Neural Underpinnings of Financial Decision Bias in Older Adults: Putative Theoretical Models and a Way to Reconcile Them

**DOI:** 10.3389/fnins.2019.00184

**Published:** 2019-03-14

**Authors:** Michael McCormick, Valerie F. Reyna, Karlene Ball, Jeffrey S. Katz, Gopikrishna Deshpande

**Affiliations:** ^1^Department of Psychology, Auburn University, Auburn, AL, United States; ^2^Human Neuroscience Institute, Cornell University, Ithaca, NY, United States; ^3^Department of Human Development, Cornell University, Ithaca, NY, United States; ^4^Center for Behavioral Economics and Decision Research, Cornell University, Ithaca, NY, United States; ^5^Magnetic Resonance Imaging Facility, Cornell University, Ithaca, NY, United States; ^6^Center for Research on Applied Gerontology, University of Alabama at Birmingham, Birmingham, AL, United States; ^7^Department of Electrical Computer Engineering, AU MRI Research Center, Auburn University, Auburn, AL, United States; ^8^Center for Neuroscience, Auburn University, Auburn, AL, United States; ^9^Alabama Advanced Imaging Consortium, Birmingham, AL, United States; ^10^Center for Health Ecology and Equity Research, Auburn University, Auburn, AL, United States

**Keywords:** decision bias, brain imaging, older adults, dual process theory, default-executive coupling hypothesis of aging (DECHA), default mode network (DMN), fronto-parietal control network, financial decision-making

Older adults face many growing challenges to their economic well-being that directly affect their autonomy and happiness. Increased medical expenses coupled with reduced mobility, impaired eyesight and hearing, and other external factors often lead older adults to retire and accept a fixed income that effectively decreases as they continue to age. This leaves less room for error and a reduced opportunity to recover from poor financial choices, such as those arising from scams and fraud of which older adults are often the target. Biological changes also challenge the decision-making processes of older adults, in particular, an older person's ability to manage personal finances (Lachs and Han, 2015). Age-related declines in the structural volume and functioning of the prefrontal cortex (PFC), altered emotion/reward processing (E-RP), and altered connectivity involving the default mode network (DMN) all play a role in decision making, but compensatory mechanisms also exist (e.g., conserved gist memory; Reyna and Brainerd, 2011). In addition, recent evidence involving the DMN has been interpreted as challenging the traditional view that biased decision making stems from E-RP (Smith et al., 2015; Li et al., 2017). These and other findings suggest an alternative framework for understanding the neural network underpinnings financial decision bias in older adults. In this review, we contrast (a) an interactive relationship such that: DMN activation/connectivity reduces resources dedicated to the cognitive control system to regulate the reward system, increasing the influence of emotion/reward sensitivity on choices and subsequently increasing decision bias with (b) an alternative account of DMN activity that adds to traditional dual-process factors by linking subjective, internal representations to the DMN and to gist-based biases. We briefly review the literature in these areas and describe PFC decline, altered E-RP, and altered DMN in aging. These processes may together affect financial decision making in older adults. We begin, however, with a brief description of decision bias and how traditional dual-process theory is used to explain such bias.

## Traditional Dual-Process View of Financial Decision Bias

The way in which individuals value options is central to the study of financial decision bias and consistently choosing options with the greatest subjective value is generally accepted as rational, even if objectively superior options are available (Von Neumann and Morgenstern, 1944). Importantly, superficial factors such as the positive or negative wording of identical options should not impact choice when outcomes remain equivalent. Irrational decision bias occurs when systematic changes in choice patterns emerge despite such equivalence (Tversky and Kahneman, 1986). These so-called “irrational” decision biases (see Reyna, 2012, 2018) might form a basis of scams and fraud attempts, leading to expected shifts in behavior for objectively unjustifiable reasons (e.g., the mere framing of options).

**Figure 1:**
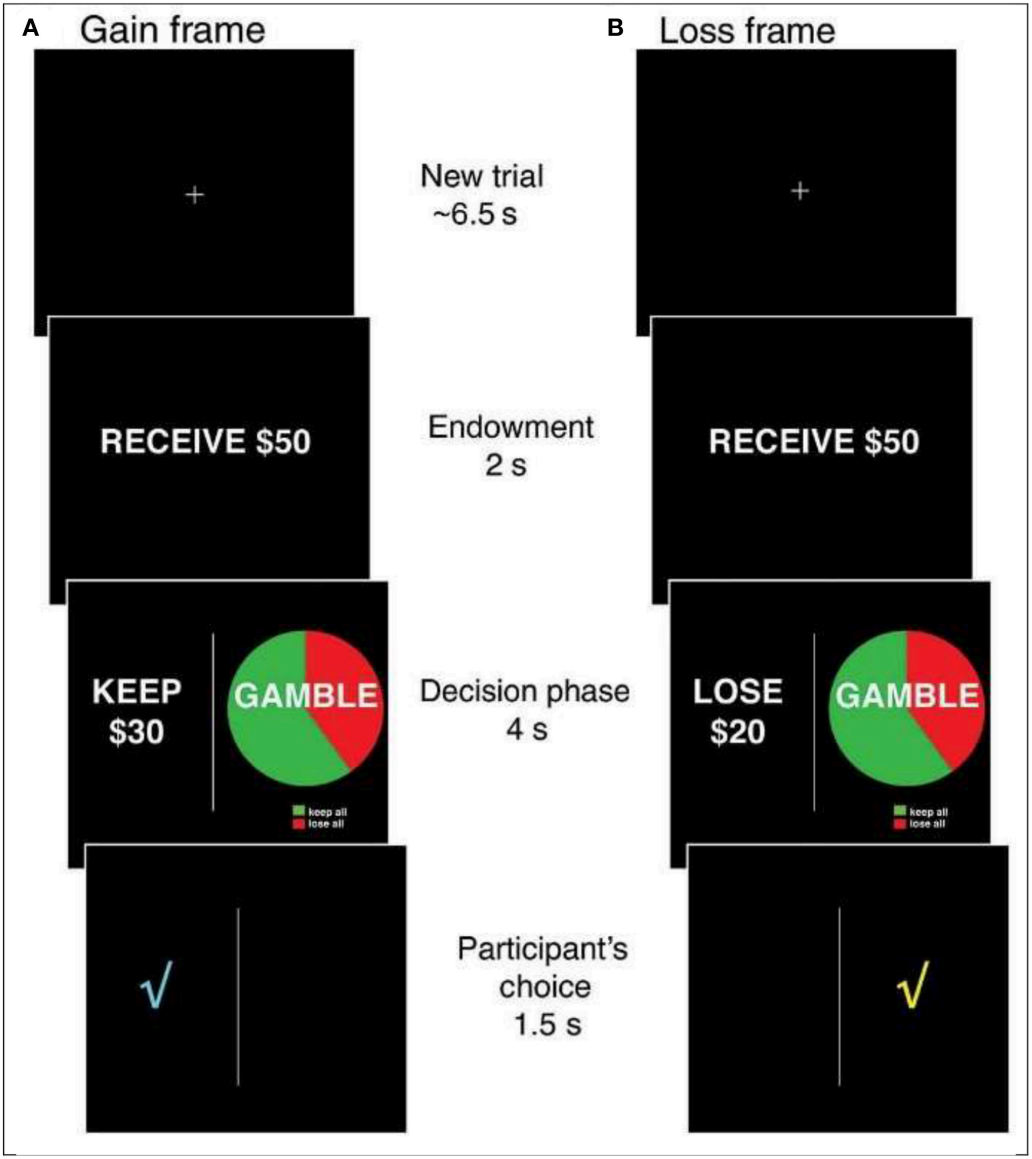
The Risky Choice Framing task. On each trial, an initial fixation cross is presented for ~6.5 s, followed by the presentation of an endowment for 2 s ($50 or $100). A decision screen is then presented for 4 s in which a safe option is always presented on the left and a risky option is always presented on the right. The safe and risky options are always equal in terms of expected value and range between 20 and 80% of the initial endowment. After participants indicate their choice, a check mark indicating the selected option is presented for 1.5 s before proceeding to the next trial. Gain **(A)** and loss **(B)** trials are pseudorandomly varied within subjects.

Specifically, one of the most extensively studied financial decision biases, and one that has been tested in multiple functional magnetic resonance imaging (fMRI) studies with younger adults is the risky choice framing effect (RCFE; e.g., Gonzalez et al., 2005). In the variation of the risky choice framing task most studied in neurological investigations, individuals are provided with an endowment (i.e., $50 or $100) and asked to make a choice between one of two options framed as either gains or losses (see Figure 1). One option is safe and offers the possibility of keeping (gains) or losing (losses) a proportion of the endowment for sure. A second, risky option is displayed using a pie chart that is identical between frames (i.e., a confounded version in which both gains and losses appear in the gamble; chances of winning might vary: 20, 40, 60, or 80% as indicated in the pie chart). Typically, the safe and risky options are equal in terms of expected value (the objective, long-term expected average return on a gamble if repeated over many trials) within and across gains and losses. Consistent preferences for risky, safe or neither option are all rational; shifting from preferring the risky option (typical in losses) to preferring the safe option (typical in gains) is not rational. That is, in many studies involving moderate-sized probabilities, most people were risk averse for gains (<50% risk-taking) and risk-seeking for losses (>50% risk-taking), displaying a framing bias. When probabilities are very small, they tend to be overweighted, producing opposite risky shifts—risk taking for gains and risk seeking for losses. These shifts in risk-taking could have dramatic effects on financial well-being in the real world and present opportunities for scams and fraud, including the selling of sub-optimal financial security services to protect against the fear of unlikely losses (e.g., expensive insurance; but see Reyna, 2018). Because financial scams and fraud involve taking risks, it is important to investigate the psychological and neural bases of RCFEs in older adults.

In regard to RCFEs and other financial decision biases, the bulk of studies have relied on one of a host of traditional dual-process theories to interpret their results. Although subtle differences exist, most of these views agree that decision bias results from a failure of deliberative thinking to regulate emotion/reward processing (E-RP; Kahneman and Frederick, 2007; Evans and Stanovich, 2013). When a choice problem is encountered, as in the case of a gain- or loss-framed monetary gamble, individuals have an initial positive or negative emotional/reward response and this response must be regulated to achieve desirable behavior. If responses are not regulated, then behavior may differ in undesirable ways, such as the tendency to select risky prospects (i.e., gambles) due to the mere wording of options. Note that while some researchers have highlighted the positive role that emotions can play in providing feedback during the decision-making process (Bechara et al., 1997; Schiebener and Brand, 2015), most traditional dual-process accounts emphasize the negative impact of emotions on decision bias, and this view typifies the majority of dual-process explanations of RCFEs.

Insufficient effort or a lack of necessary “mindware” (i.e., learned decision rules) have been thought to account for failures of cognitive control processes to regulate E-RP, but enhanced E-RP may also lead to regulatory failures by increasing regulatory demands beyond the limiting abilities of cognitive control systems. When effort and ability are sufficient, however, control network processing (i.e., deliberative thinking) exerts a top-down regulating influence on subjective emotional/reward responses and choices are rational, consistent, and free from decision bias. Thus, most traditional dual-process theories view decision making as a competition between E-RP and control processes.

In this view, financial decision bias could represent a failure of the PFC centered control network to properly modulate E-RP activity in the valuation network. Neural structures associated with E-RP include the amygdala and nucleus accumbens as well as medial portions of the PFC. Thus, medial portions of the PFC, in close physical proximity to the control network, are also implicated in reward network processing (Hare et al., 2009), and as will be discussed, this region also appears in common definitions of the DMN. This observation may be important for understanding how the “compensation” strategies used by older adults (discussed below) leads to undesirable co-activation between neural networks and an increase in financial decision bias. Notwithstanding, a voluminous body of research has provided general support for the traditional dual-process view, including the neurological evidence described in the preceding paragraph.

## PFC Decline and Enhanced E-RP Support the Traditional Dual-Process View

Many changes in the decision making of older adults may be related to declines in prefrontal brain regions. Known as the “frontal lobe” hypothesis (Moscovitch and Winocur, 1995; West, 1996), there is large agreement in the neuroscience literature that one of the most pervasive effects of normal aging on the brain is a gradual decline in structural volume and the physical connections between brain regions (see, Weller et al., 2011; Samanez-Larkin and Knutson, 2015 for reviews). That is, as individuals age, the physical connections (i.e., structural connectivity) that underlie functional network connectivity begin to deteriorate, compromising the structural integrity of networks and their functioning. In particular, frontal regions implicated in reward valuation and cognitive control decline most rapidly with age and are associated with impairments in decision making (see Samanez-Larkin and Knutson, 2015; Koestner et al., 2016 for reviews). Relevant frontal structures associated with reward valuation include the ventromedial prefrontal cortex (vmPFC); structures involved in control network processing include dorsolateral portions of the parietal and frontal cortices (i.e., the fronto-parietal control network; FPCN) as well as portions of the dorsomedial prefrontal cortex (dmPFC) in close physical proximity to reward network structures (Fox et al., 2005; Fair et al., 2009; Anderson et al., 2011; Power et al., 2011; Yeo et al., 2011; Barber et al., 2013; Sato et al., 2015; Smith et al., 2015; Li et al., 2017).

These findings are relevant for traditional “dual process” theories of decision making which, as noted above, hold that decision bias occurs when control network processing fails to regulate E-RP (De Martino et al., 2006). More precisely, according to traditional dual-process theories, processing in emotion/reward regions (amygdala, vmPFC) is inhibited by processing in cognitive control regions (FPCN; dmPFC) to resist decision bias. Structural declines in the brains of older adults, however, are thought to affect the competition between control and emotion/reward network processing. Specifically, functional changes are thought to overlay these structural declines and result in impaired reward processing and a reduced ability to integrate options (Samanez-Larkin and Knutson, 2015).

In regard to behavioral regulation, functional declines in prefrontal control network regions may reduce the ability of some older adults to predict and appropriately exert control over responses to emotions as well as rewards/valuations. In terms of neural network interactions, this reduced ability to modulate emotion/reward network processing could manifest as a reduction in the anti-correlation in neural activity between networks. That is, activity in the control network would be negatively (i.e., anti) correlated with activity in the emotion/reward network for the control network to regulate E-RP and resist financial decision bias, and functional declines in control network processing would reduce the ability of the control network to establish and maintain this negative correlation. As a result, the anti-correlation between the control and emotion/reward networks should be greater in younger vs. older adults, according to the traditional view.

Alternatively, behavioral regulation (i.e., resistance to financial decision bias) may be negatively affected in older adults primarily due to increases in E-RP. A number of exceptions have been reported, but the bulk of evidence suggests that older adults have a positivity bias (Carstensen et al., 1999; Mather and Carstensen, 2003; Adams, 2004; Leigland et al., 2004; Dudley and Multhaup, 2005; Löckenhoff and Carstensen, 2008), are sensitive to rewards (Bauer et al., 2013), are less responsive to and have poorer memory for negative stimuli (for review see Samanez-Larkin and Knutson, 2015), often base decisions on affect more than the numeric value of options, particularly when decisions are complex and require the integration of multiple options (Eppinger et al., 2013; Worthy et al., 2016), and generally rely more on the use of heuristics and biases (Löckenhoff and Carstensen, 2008; Worthy and Maddox, 2012; Worthy et al., 2016). However, it should be noted that reliance on memory for gist, as opposed to precise details such as numbers, occurs in adults regardless of whether decisions are complex (Reyna and Brainerd, 2011; Reyna et al., 2014). Moreover, memory for gist is relatively conserved in healthy aging. Thus, it is wrong to say that “memory” declines in old age; instead, verbatim memory declines creating greater reliance on relatively intact gist memory (Reyna, 2011).

Perhaps not surprisingly then, some research has shown that older adults are more susceptible to framing manipulations that vary the positive or negative wording of objectively equivalent options, although this may be driven primarily by differences in the domain of gains (Weller et al., 2011; see Best and Charness, 2015 and Mata et al., 2011, for reviews). In a recent study (Weller et al., 2011), a large sample of individuals aged 5–85 completed a form of risky choice framing task known as the “Cups” task. Overall, whereas older and younger adults displayed similar levels of risk-taking for losses, older adults took fewer risks in the domain of gains. Other research appears to corroborate this finding, but several exceptions have also been reported (see Best and Charness, 2015 and Mata et al., 2011, for reviews). In order to clarify these age differences, it is crucial in future research to unconfound factors that are known to influence choices, such as risk preferences for framing problems and verbatim memory for outcomes of prior gambles (i.e., older and younger adults are not solving the same decision problems if they remember prior relevant outcomes differently).

## Compensatory Activation Improves Task Performance but May Have Undesirable Consequences

Despite claims of overall greater susceptibility to financial decision bias among some older adults, it should be noted that many are able to achieve performance levels comparable to younger adults by recruiting compensatory activation in additional brain regions (Halfmann et al., 2014; Lighthall et al., 2014; Worthy et al., 2016). Termed the “compensation hypothesis,” it is thought that older adults are sometimes able to compensate for structural declines and achieve performance levels similar to younger adults by recruiting additional resources and/or employing alternative decision strategies, such as an increase in the use of gist-based heuristics (Mata et al., 2011; Worthy and Maddox, 2012; Halfmann et al., 2014; Lighthall et al., 2014; Worthy et al., 2016). Indeed, experiments and mathematical models have shown that older adults compensate in recall tasks by using gist memory to reconstruct verbatim items that were studied (Reyna, 2011). When reported in neurological investigations, compensation in older adults is often inferred from the presence of bilateral activation in the dlPFC (which is part of the FPCN) whereas only unilateral activation is observed in younger adults, indicating that different brain regions are recruited in older adults to compensate for age-related declines.

Another key test is based on task performance–if performance increases in concert with the recruitment of additional areas and/or resources, then recruitment was compensatory; if not, then the additional resources/activations were not compensatory (Lighthall et al., 2014). Recent neuroimaging studies have supported the compensation hypothesis (Halfmann et al., 2014; Lighthall et al., 2014; Worthy et al., 2016). In one, right dlPFC and striatal activation significantly correlated with performance on a reward learning task in older adults (*r* = 0.66, *p* < 0.01), but not in younger adults (*r* = 0.17, *p* = 0.49), consistent with the notion that increased activation in the DLPFC compensated for age-related declines in dlPFC functioning (Worthy et al., 2016). These findings shed light on the compensation strategies used by older adults and suggest that preserved (and possibly extended) control network functionality may overcome structural declines and facilitate resistance to financial decision bias.

One issue that may arise from compensatory activation, however, is the extent to which regions outside of the control network, but located in close spatial proximity to the control network, may be recruited. A case in point are the lateral and medial PFC regions of the control network and medial PFC regions of the emotion/reward network. In younger adults, control network and emotion/reward network processing are typically uncorrelated at rest and become more anti-correlated as the control network exerts a regulatory influence on E-RP. This is an important condition in resisting decision bias according to the traditional dual-process view.

Co-activation with other neural networks may also affect financial decision bias. Although cognitive control processes modulate framing biases, such processes are not necessary either to generate or to eliminate framing biases (Kühberger and Tanner, 2010; Reyna et al., 2014). Instead, reliance on simple gist representations (e.g., preferring to gain some money for sure over the possibility in the risky option of some money or no money) generates framing biases and reliance on precise verbatim process eliminates those biases. These effects occur without varying cognitive control, although when cognitive control does vary, framing biases can be modified (see Stanovich and West, 2008). In addition, recent evidence suggests that DMN activation is associated with susceptibility to financial decision bias, and portions of the medial PFC are also included in this network (Smith et al., 2015; Li et al., 2017). The DMN reflects more than an absence of task engagement (Smith et al., 2015). It also reflects internal mental representations, such as the gist memory representations that underlie framing biases (see Reyna and Huettel, 2014).

## DMN Activation Affects Decision Bias in Younger Adults and Is Enhanced in Older Adults

Despite voluminous behavioral and some neurological support, a growing body of research calls into question the completeness of the traditional dual-process view of decision making (Gonzalez et al., 2005; Kruglanski et al., 2006; O'Keefe and Jensen, 2007, 2009; Kühberger and Tanner, 2010; Gigerenzer and Gaissmaier, 2011; Reyna and Brainerd, 2011; Wright et al., 2012, 2013; Mega et al., 2015; Smith et al., 2015; Van't Reit et al., 2016; Li et al., 2017; Seta et al., 2017; Voss et al., 2018). Two recent brain imaging studies, for example, reported that increased DMN activation (and/or connectivity) was related to RCFE susceptibility to a greater extent than reward related processing, contrary to the predictions of traditional dual-process theories (Smith et al., 2015; Li et al., 2017). The DMN is comprised, in part, of the posterior cingulate cortex (PCC), the medial prefrontal cortex (mPFC) and the inferior parietal lobule (IPL), and has been associated with both internal/subjective thought processing and task disengagement (Turner and Spreng, 2015). Note that a portion of the mPFC is included in definitions of the DMN. Thus, distinct but physically close portions of the mPFC belong to the DMN, the control network and the emotion/reward network.

In one study which was conducted with younger adults, a task-based connectivity analysis (i.e., Psychophysiological Interaction or PPI) revealed that RCFE susceptibility (when participants received feedback) was associated with increased coupling between the DMN and a region within the mPFC which is not part of the DMN (Smith et al., 2015). Resistance to the RCFE also included activation in the mPFC, but in a different portion and as part of an Executive Control network (EC) that overlaps with the FPCN. Thus, distinct portions of the mPFC were associated with susceptibility and resistance to the RCFE, dependent on functional connections with the DMN and EC, respectively. In another study, DMN activation was associated with RCFE susceptibility while resistance to the RCFE was associated with neural profiles related to task engagement (Li et al., 2017). The task engagement related neural profiles were generated using the online database “Neurosynth” and contained frontal and parietal regions associated with cognitive control. Other evidence suggests that DMN activation/connectivity is likely to be enhanced relative to cognitive control or task engagement in older adults, further challenging the ability to resist financial decision bias (Turner and Spreng, 2015).

Importantly, both framing studies found that susceptibility to financial decision bias was primarily the result of DMN activation/connectivity and not the result of activation/connectivity in the amygdala, part of an emotion/reward network. Accordingly, findings for both were interpreted as inconsistent with the traditional dual-process view. In the case of Li et al. (2017), DMN involvement was interpreted as reflecting task disengagement, with the implication that decision bias arises from a lack of task involvement. However, Smith et al. (2015) interpreted DMN activation as reflecting “interoceptive” attentional processes. Note that task disengagement—by itself—cannot produce framing biases *per se*; simply disengaging produces random responses. Thus, it is reasonable to conclude that processes in the DMN, in concert with other brain areas, give rise to a behavioral framing effect.

## Default-Executive Coupling Hypothesis of Aging (DECHA)

A relatively new model known as the Default-Executive Coupling Hypothesis of Aging (DECHA) is consistent with some results we have discussed and may help identify a second mechanism through which decision bias increases in older adults (Turner and Spreng, 2015). According to the DECHA, due to prefrontal structural decline, older adults have a reduced ability to modulate prefrontal activity involving the EC (primarily dorsolateral regions associated with the FPCN) and this co-occurs with reduced suppression of the DMN. Reduced suppression of the DMN produces relatively higher levels of DMN activation/connectivity during external task completion than normally observed in younger adults. The end result is that the EC and DMN tend to be co-activated (i.e., functionally connected) to a greater extent in older than younger adults, supporting a greater reliance on internal mental representations and on experience when making decisions. That is, increased EC-DMN coupling is seen as reflecting the increased active incorporation of subjective/internal thoughts into the task-relevant processing of the EC.

In regard to financial risk taking, increased EC-DMN coupling may increase the extent to which older adults consider the subjective, personal consequences of their choices relative to younger adults. This increased internal processing may increase the likelihood that older adults consider the gist that “some money will be lost” or “some money will be saved,” and to the extent that they value saving some money over saving none, they would be expected to display risk-aversion when considering gains and risk-seeking behavior when considering losses (Reyna and Brainerd, 2011). In addition to enhanced EC-DMN coupling among older vs. younger adults, the extent of EC-DMN coupling may be associated with bias susceptibility. If so, resistance to financial decision bias would be associated with preserved EC-DMN segregation. Future research is needed to test these predictions but such findings would have important theoretical implications as they would suggest that the traditional dual process view is incomplete or inaccurate regarding the processes underlying decision making in older adults.

It is important to note, however, that the findings discussed above were all derived using task-based data; no previous studies have reported a link between framing susceptibility and DMN connectivity in resting-state data. This is an important gap in the literature given that it is difficult to completely infer the operation of intrinsic functional networks when a task is being completed. That is, task-based data reveals activation in areas that are used for task completion, but it is probably not appropriate to infer that the same areas would co-activate or form a network when a different task is completed or in a baseline state when the subject is not engaged in an active task. This is important because resting state baseline activity is known to bias or modulate task-evoked activity (Yeo et al., 2011). Only by examining resting-state data when no task is required is it possible to infer the fundamental organization of the brain and measure the extent to which intrinsic functional networks affect behavior (Yeo et al., 2011; see also Mesulam, 1990 and Posner et al., 1988, for early discussions). Thus, the analysis of resting-state data is important for examining the theoretical neural underpinnings of decision making. Moreover, detecting susceptibility to decision bias in resting-state scans would be highly beneficial to society given that such scans are relatively low-cost and patient friendly, requiring only that participants lie still for a few minutes.

## Potential Interactions and Reconciliation of Findings

Given the persistence and ubiquity of traditional dual-process distinctions, despite disconfirmatory evidence, it is important for future research to reconcile how representations of options, reward/emotion and cognitive control (or executive processes) may together significantly affect financial decision bias among older adults. In aging, PFC decline and enhanced E-RP could provide one source of upward pressure on the magnitude of decision bias, and enhanced DMN activation/connectivity could provide another source of bias. If so, individuals could display susceptibility to decision bias due to either PFC decline (with or without changes in E-RP) or enhanced DMN activation/connectivity, or both.

Limited resources and capacity constraints may restrict the functioning of these systems and may account for their interaction (though see Reyna and Brainerd, 1995, for contradictory evidence across the lifespan). Note that if enhanced DMN activation/connectivity taps internal thought processing of gist, this reduces the need for cognitive resources and inhibition associated with cognitive control regions (Reyna and Rivers, 2008). Hence, there is less need to regulate E-RP. Nevertheless, a lack of sufficient deactivation of the DMN during task performance has been implicated in sub-optimal or even maladaptive behavioral performance in other contexts (see Turner and Spreng, 2015). Therefore, it is crucial to test the applicability of these mechanisms in the context of financial decision bias in older adults.

In order to test which of the possibilities described above may better reconcile the traditional dual process and DMN-centered approaches to financial decision bias in older adults, one may employ advanced connectivity models (in additional to traditional activation) for investigating the interaction between control, reward, emotion, and DMN regions. A straightforward choice will be either PPI (task-based; Di and Biswal, 2018) or Pearson's correlation based functional connectivity (resting state; Rangaprakash et al., 2017). ICA or other alternative methods could also be used to characterize resting state functional connectivity (Rangaprakash et al., 2013; Syed et al., 2017). However, these models are incapable of identifying the directionality of interactions. Therefore, we propose using a data-driven method such as multivariate autoregressive model coupled with hemodynamic deconvolution to determine candidate models (Havlicek et al., 2011; Deshpande et al., 2012; Sathian et al., 2013; Hutcheson et al., 2015; Sreenivasan et al., 2015; Rangaprakash et al., 2018). This approach has been used in the context of both task-based and resting state data (Grant et al., 2014, 2015; Liang et al., 2014, 2016; Wheelock et al., 2014; Wang et al., 2017; Zhao et al., 2017; Palaniyappan et al., 2018). Such candidate models can then be tested using a confirmatory approach such as Dynamic Causal Modeling (DCM; deterministic DCM for task-based and stochastic/spectral DCM for resting state; Friston et al., 2003; Razi et al., 2017). Multiple regression analyses could be used to determine which models more accurately describe how the brain makes choices and becomes susceptible to financial decision bias.

## Conclusion

Age-related declines in brain functioning accelerate after age 60 and may contribute to the tendency of some older adults to be prone to financial decision bias. Regardless of whether there are age differences in financial bias, it is unarguable that the impact of poor choices is greater for older than younger adults because they are past their peak earning years. Poor economic choices arising from cognitive changes not only affect individual older adults, they may also increase societal costs, such as reliance on federal programs. In any case, understanding the factors that increase suffering in society, such as financial decision biases in older adults, is important for all individuals and the society at large. Traditional dual-process theory is most commonly used to explain susceptibility to decision bias and has received considerable empirical support, but recent reports suggest that this view is either inaccurate or incomplete. While the traditional view holds that susceptibility to decision bias results from a failure of PFC-centered cognitive control processes to regulate reward processing, more recent evidence suggests that DMN activation/connectivity may play a bigger role by introducing subjective/internal thoughts into the decision process.

Of course, PFC decline and enhanced DMN activity/connectivity may both contribute to decision bias in older adults. We propose a framework to test whether these factors operate independently or jointly, thereby providing a means for reconciling competing accounts. The joint influence of these factors may be observed if increased EC-DMN (or FPCN-DMN) connectivity among older adults is found to reduce the resources or capacity available to the EC (or FPCN) to control emotion/reward network processing, and this results in increased decision bias. If so, then susceptibility to the RCFE should be associated with a failure of the EC to control reward/emotion processing, but this relationship should be mediated by enhanced coupling between the EC and DMN. Future research will be needed to test these predictions, particularly studies involving resting-state connectivity analyses that investigate the fundamental organization of the brain and are not influenced by the particular features of a single task.

## Author Contributions

All authors contributed their opinion to this opinion manuscript. MM primarily wrote the manuscript based on his understanding of the collective opinion of all authors while others edited them to reflect their opinions accurately.

### Conflict of Interest Statement

The authors declare that the research was conducted in the absence of any commercial or financial relationships that could be construed as a potential conflict of interest.
